# Leveraging spreadsheet analysis tool for electrically actuated start-up flow of non-Newtonian fluid in small-scale systems

**DOI:** 10.1038/s41598-022-24287-2

**Published:** 2022-11-21

**Authors:** Manideep Roy, Pritam Chakraborty, Pranab Kumar Mondal, Somchai Wongwises

**Affiliations:** 1 Department of Mechanical Engineering, National Institute of Technology Durgapur, Durgapur, 713209 India; 2 Microfluidics and Microscale Transport Processes Laboratory, Department of Mechanical Engineering, Indian Institute of Technology Guwahati, Guwahati, 781039 India; 3 Department of Mechanical Engineering, Faculty of Engineering, King Mongkut’s University of Technology Thonburi (KMUTT), Bangkok, 10140 Thailand; 4 National Science and Technology Development Agency (NSTDA), Pathum Thani, 12120 Thailand

**Keywords:** Engineering, Mathematics and computing

## Abstract

In this article, we demonstrate the solution methodology of start-up electrokinetic flow of non-Newtonian fluids in a microfluidic channel having square cross-section using Spreadsheet analysis tool. In order to incorporate the rheology of the non-Newtonian fluids, we take into consideration the Ostwald-de Waele power law model. By making a comprehensive discussion on the implementation details of the discretized form of the transport equations in Spreadsheet analysis tool, and establishing the analytical solution for a special case of the start-up flow, we compare the results both during initial transience as well as in case of steady-state scenario. Also, to substantiate the efficacy of the proposed spreadsheet analysis in addressing the detailed flow physics of rheological fluids, we verify the results for several cases with the corresponding numerical results. It is found that the solution obtained from the Spreadsheet analysis is in good agreement with the numerical results—a finding supporting spreadsheet analysis's suitability for capturing the fine details of microscale flows. We strongly believe that our analysis study will open up a new research scope in simulating microscale transport process of non-Newtonian fluids in the framework of cost-effective and non-time consuming manner.

## Introduction

With the advent of the Lab-On-a-Chip (LOC) devices/systems, typically find practical relevance in biomedical applications, biochemical processes, medical diagnostics and digital microfluidics, transportation of small fluid volume alongside embarking on several fluidic functionalities in these devices has become a great topic of research to the microfluidics community^1–3^. The underlying flow dynamics at microfluidic scale, however, is greatly influenced by the surface characteristics of the bounding substrate, and at times, is intricately governed by the solid–liquid interfacial interactions^4,5^. A substantial amount of practical applications together with the intense interests of achieving augmented fluidic functionalities in microflows have compelled researchers towards better understanding of the field driven flow dynamics, i.e., electrically actuated microscale transport, flow manipulations using applied magnetic field, thermocapillarity induced flow etc.^4,6–8^. Researchers have also studied the effect of Joule heating and thermal radiation on the thermal transport characteristics of heat in the purview of electroosmotic flow for both Newtonian and power law fluids considering different properties of the channel wall^9–11^. It is worth mentioning here that several intricate features associated with the field driven transport in microfluidic channels have been addressed by the researchers either from the paradigm of experimental investigations^12–14^ or from theoretical perspectives^15^. Despite this subject being studied over the past few years, an analysis of the microscale start-up flow which finds relevance in aforementioned applications^16,17^, in the presence of externally applied effects like electric and magnetic field etc., using an analytical framework is of significant practical interest, attributed primarily to the non-involvement of expensive and time consuming numerical methods.

In most of the applications, as mentioned above, the working fluid exhibits non-Newtonian rheological behaviour. The momentum equations governing the flow dynamics of non-Newtonian fluids are, however, contain the non-linear diffusion terms^18–24^. Existence of the non-linear diffusion terms in the momentum equations makes these equations analytically intractable essentially for obtaining the desired solutions^15,18,25,26^. The unsteady flow scenario brings about even more complexity to the solution process of transport equations in analytical framework. Very often, these equations, which are integrated with the non-linear constitutive behaviour, are solved numerically, and the solution process becomes cost ineffective and time consuming as well, even in the paradigm of microscale transport (typically, known as low Reynolds number transport). In this aspect, the spreadsheet tool of Microsoft excel has successfully been used to obtain the solutions for a variety of flow problems, including external fields^16,27^. Quite notable, as observed from the reported analysis, the spreadsheet analysis tool can effectively capture the detailed flow physics of interest at microfluidic scale^16,17^.

Here, we discuss about the applicability of spreadsheet tool in solving the start-up flow of inelastic non-Newtonian fluid in a microfluidic channel, which is considered to have square cross-section, in the presence of an applied electric field. We, first, scrutinize the capability of spreadsheet tool from the perspective of electrostatics essentially to obtain the potential field. Following this, we look for the solution of momentum transport equations to get the velocity field. In contrast to the conventional methods available in this paradigm, our analysis, consistent with the spreadsheet tool, is found to provide reliable results taking the effect of electrical forcing into account. In particular, we have been able to establish that the proposed tool can successfully capture the effects due to electrical double layer phenomenon on the underlying transport quite accurately even for the non-Newtonian fluids at small scale. The tool can of huge interest to the researcher where commercial software is not readily available for solving the complex transport equations governing the flow dynamics of non-Newtonian fluids.

## Discussion of the problem: definition and geometry

**Figure 1:**
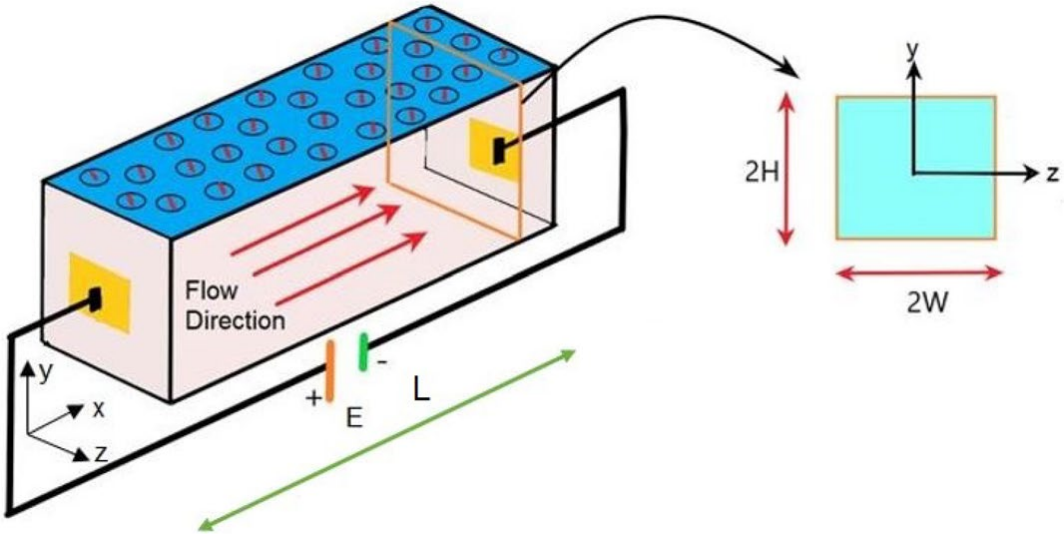
Plots depict the schematic view of the microfluidic channel that is considered in this study. The channel dimensions with the coordinate system, are shown with the zoomed-in view of the *y* − *z* cross-section.

For the present study, we consider a non-accelerating, isothermal, unsteady flow of a non-Newtonian fluid through a rectangular (square) microchannel as schematically shown in Fig. 1. To incorporate the rheology of the homogeneous, isotropic and non-Newtonian fluid, we consider the Ostwald–de Waele power law model in this analysis^23,28^. The dimensions of the fluidic channel chosen for this analysis are as follows: height i.e., the distance between two plates is $2H$, width is $2W$ and length is $L$. We consider that the width ($2W$) and height ($2H$) of the channel are of the same order, whereas the length of the channel is considered to be much larger than its other two dimensions ($L > > 2H \sim 2W$). The combined effect of the electroosmotic force and applied pressure gradient drive the flow through the channel as considered in this study. To realise the effect of electroosmosis in the flow process, we consider that the walls of the microchannel to bear a net charge, which further gives rise to the generation of Electrical Double Layer (EDL) in contact with the ionic solution. It is because of the formation of EDL, a potential is developed therein and, an electric field $E(E_{x} ,0,0)$ applied externally when interacts with the induced potential developed inside the EDL, provides the flow with a driving force. Also, we consider the flow to be unidirectional $u(u,0,0)$ for the present study, while we look out for the solution of flow velocity and electrostatics in the 2-D rectangular cross-section (YZ plane) as shown in Fig. 1. It may be mentioned here that the Ostwald–de Waele power law model is used by many researchers in investigating the underlying transport features of inelastic non-Newtonian fluid even under the influence of electrical forcing^28,29^. It is also assumed that the thermal and the physical properties of the fluid chosen in this study are constant.

## Mathematical formulation

The Poisson–Boltzmann equation for the underlying electrostatics and the Cauchy momentum transport equation for the description of flow velocity dictate the flow dynamics for the case under consideration in this analysis. It is needless to mention here that the continuity equation needs to be solved together with the aforementioned equations essentially to satisfy the conservation of mass constraint in the flow field. In the succeeding sections, we discuss the pertinent equations and the associated conditions of the boundary both in their dimensionless and dimensional forms. Following the Ostwald–de Waele power law model, the constitutive equation can be written as: ${{\varvec{\uptau}}} = m\left( {\dot{\gamma }} \right)^{n}$, where $m$ is the index of flow consistency and $n$ is the is the index of blow behaviour^23,29,30^. It is worth mentioning here that $\dot{\gamma }$ is the tensor for strain rate and is given as $\dot{\gamma } = \frac{1}{2}\left[ {e_{ij} :e_{ij} } \right]^{{{1 \mathord{\left/ {\vphantom {1 2}} \right. \kern-\nulldelimiterspace} 2}}}$, where $e_{ij}$ is the strain rate tensor^28,29^.

### Poisson Boltzmann equation

The Poisson Boltzmann equation considering negligible variation of the ionic species in the axial direction is given as:

1 $$\frac{{\partial {}^{2}\psi }}{{\partial y{}^{2}}} + \frac{{\partial {}^{2}\psi }}{{\partial z{}^{2}}} = \kappa^{2} \psi .$$

Here, $$\psi$$ is the electric potential induced, $$\kappa \left( { = \left[ {{{\left( {2z^{2} e^{2} n_{0} } \right)} \mathord{\left/ {\vphantom {{\left( {2z^{2} e^{2} n_{0} } \right)} {\left( {\varepsilon_{0} \varepsilon_{r} k_{B} T} \right)}}} \right. \kern-\nulldelimiterspace} {\left( {\varepsilon_{0} \varepsilon_{r} k_{B} T} \right)}}} \right]^{{{\raise0.7ex\hbox{$1$} \!\mathord{\left/ {\vphantom {1 2}}\right.\kern-\nulldelimiterspace} \!\lower0.7ex\hbox{$2$}}}} } \right)$$ is the parameter for Debye–Hückel linearization for the electrolyte layer, $$z$$ is the valence of ions, $$\varepsilon_{0}$$ is the permittivity of the free space, $$e$$ is the protonic charge, $$\varepsilon_{r}$$ is the relative permittivity, $$k_{B}$$ is the Boltzmann constant, $$n_{0}$$ is the neutral charge density, and $$T$$ is the absolute temperature.

In order to obtain the potential distribution, the following boundary conditions are used to solve Eq. (1) and given as:

Specified zeta potential at the wall of the microchannel and symmetry at the center:

2 $$\left. \psi \right|_{y = \pm H} = \zeta \;\;\; \text{and} \;\;\; \left. {{{\partial \psi } \mathord{\left/ {\vphantom {{\partial \psi } {\partial y}}} \right. \kern-\nulldelimiterspace} {\partial y}}} \right|_{y = 0} = 0.$$

Here, we incorporate the dimensionless quantities: $\overline{\psi } = {\psi \mathord{\left/ {\vphantom {\psi \zeta }} \right. \kern-\nulldelimiterspace} \zeta }$, $\overline{y} = {y \mathord{\left/ {\vphantom {y H}} \right. \kern-\nulldelimiterspace} H}$, $\overline{z} = {z \mathord{\left/ {\vphantom {z H}} \right. \kern-\nulldelimiterspace} H}$, $\overline{\kappa } = \kappa H$ and $\zeta = {{k_{B} T} \mathord{\left/ {\vphantom {{k_{B} T} {ze}}} \right. \kern-\nulldelimiterspace} {ze}}$ to make Eq. (1) and the associated boundary conditions dimensionless. The dimensionless form of Eq. (1) and the associated boundary conditions [Eq. (2)] reads as:

3 $$\left. \begin{gathered} \left( {\frac{{\partial {}^{2}\overline{\psi }}}{{\partial \overline{y}{}^{2}}} + \frac{{\partial {}^{2}\overline{\psi }}}{{\partial \overline{z}{}^{2}}}} \right) = \overline{\kappa }^{2} \overline{\psi } \hfill \\ \left. {\;\overline{\psi }} \right|_{{\overline{y} = \pm 1}} = - 1\;\;\;{\text{and}} \;\;\; \left. {{{\partial \overline{\psi }} \mathord{\left/ {\vphantom {{\partial \overline{\psi }} {\partial \overline{y}}}} \right. \kern-\nulldelimiterspace} {\partial \overline{y}}}} \right|_{{\overline{y} = 0}} = 0 \hfill \\ \end{gathered} \right\}.$$

### Discretization of non-dimensional potential distribution equation

We shall represent as well as compare the results of this analysis, obtained from both spreadsheet analysis and numerical method in “Results and discussion”. We shall take this endeavour essentially to ascertain the applicability of the spreadsheet analysis tool in capturing the detailed flow physics of our interest. Since we look for the depiction of numerical results obtained from a scheme consistent with finite difference methods as well^26^, it would be more appealing to discuss about the discretization of the transport equations. Accordingly, here we write the discretized form of Eq. (3), used for obtaining the numerical solutions. By appealing to the central difference second-order discretization scheme for space, we get the following expression for potential.

4 $$\overline{\psi }_{(i,j)} = {{\left( {\frac{{\overline{\psi }_{(i + 1,j)} + \overline{\psi }_{(i - 1,j)} }}{{\Delta \overline{y}^{2} }} + \frac{{\overline{\psi }_{(i,j + 1)} + \overline{\psi }_{(i,j - 1)} }}{{\Delta \overline{z}^{2} }}} \right)} \mathord{\left/ {\vphantom {{\left( {\frac{{\overline{\psi }_{(i + 1,j)} + \overline{\psi }_{(i - 1,j)} }}{{\Delta \overline{y}^{2} }} + \frac{{\overline{\psi }_{(i,j + 1)} + \overline{\psi }_{(i,j - 1)} }}{{\Delta \overline{z}^{2} }}} \right)} {\left( {\overline{\kappa }^{2} + \frac{2}{{\Delta \overline{y}^{2} }} + \frac{2}{{\Delta \overline{z}^{2} }}} \right)}}} \right. \kern-\nulldelimiterspace} {\left( {\overline{\kappa }^{2} + \frac{2}{{\Delta \overline{y}^{2} }} + \frac{2}{{\Delta \overline{z}^{2} }}} \right)}}.$$

Here, we consider a very unique case of equal width and height for this analysis. Also, we consider uniform grid sizes i.e., $\Delta \overline{z}^{2} = \Delta \overline{y}^{2} = h_{yz}$. Considering this aspect, Eq. (4) reduces to the following form.

5 $$\overline{\psi }_{(i,j)} = \left( {\overline{\psi }_{(i,j - 1)} + \overline{\psi }_{(i - 1,j)} + \overline{\psi }_{(i,j + 1)} + \overline{\psi }_{(i + 1,j)} } \right)/\left( {\overline{\kappa }^{2} h^{2}_{yz} + 4} \right).$$

### Momentum transport equation

The Cauchy momentum equation describing the fully developed flow of a non-Newtonian power law fluid in the chosen fluidic configuration is given as:

6 $$\rho \frac{\partial u}{{\partial t}} = - \frac{\partial p}{{\partial x}} + \frac{\partial }{\partial y}\left[ {m\left( {\left( {\frac{\partial u}{{\partial y}}} \right)^{2} + \left( {\frac{\partial u}{{\partial z}}} \right)^{2} } \right)^{{\frac{n - 1}{2}}}} \right] \left( {\frac{\partial u}{{\partial y}}} \right) + \frac{\partial }{\partial z}\left[ {m\left( {\left( {\frac{\partial u}{{\partial y}}} \right)^{2} + \left( {\frac{\partial u}{{\partial z}}} \right)^{2} } \right)^{{\frac{n - 1}{2}}}} \right] \left( {\frac{\partial u}{{\partial z}}} \right) - \in_{r} \in_{0} \kappa^{2} \psi E_{x} .$$

In Eq. (6), p is the pressure, and $\rho$ is the density of the fluid. It is worth adding here that pertaining to the power law model, $\mu_{app} \left( { = m\dot{\gamma }^{n - 1} } \right)$ is the apparent dynamic viscosity of the fluid^29^. Other parameters appearing in Eq. (6) are already defined in “Poisson Boltzmann equation”. Note that the expression $\left[ {m\left( {\left( {\frac{\partial u}{{\partial y}}} \right)^{2} + \left( {\frac{\partial u}{{\partial z}}} \right)^{2} } \right)^{{\frac{n - 1}{2}}}} \right]$ in Eq. (6) accounts for the apparent viscosity $\left( {\mu_{app} } \right)$ of the fluid for the flow configuration considered in this analysis.

For the description of hydrodynamics of the present problem, we use the no slip i.e., zero velocity condition at the walls of the channel and velocity symmetry at the channel centre while solving Eq. (6).

7 $$\left. \begin{gathered} {\text{No}} \,{\text{slip}}\,{\text{condition}}:\;u\left( {y = \pm H,z = \pm W} \right) = 0 \hfill \\ {\text{Velocity}} \;{\text{symmetry}}:{{\partial u} \mathord{\left/ {\vphantom {{\partial u} {\partial y\left( {y = 0,z = 0} \right)}}} \right. \kern-\nulldelimiterspace} {\partial y\left( {y = 0,z = 0} \right)}} = 0 \hfill \\ \end{gathered} \right\}.$$

In an attempt to write Eq. (6) in its dimensionless counterpart, we use the Helmholtz–Smoluchowski velocity $U_{HS} \left[ { = n\kappa^{(1 - n)/n} \left( {\frac{{ - \varepsilon_{r} \varepsilon_{0} k_{B} TE_{x} }}{mze}} \right)^{1/n} } \right]$ as the reference velocity, while half height of the channel is taken as the reference length scale. By using these reference scales, we obtain the dimensionless form of Eq. (6) as written below.

8 $${\text{Re}} \frac{{\partial \overline{u}}}{{\partial \overline{t}}} = - 2\Gamma + \frac{\partial }{{\partial \overline{y}}}\left[ {\left( {\frac{{\partial \overline{u}}}{{\partial \overline{y}}}} \right)^{2} + \left( {\frac{{\partial \overline{u}}}{{\partial \overline{z}}}} \right)^{2} } \right]^{{\frac{n - 1}{2}}} \left( {\frac{{\partial \overline{u}}}{{\partial \overline{y}}}} \right) + \frac{\partial }{{\partial \overline{z}}}\left[ {\left( {\frac{{\partial \overline{u}}}{{\partial \overline{y}}}} \right)^{2} + \left( {\frac{{\partial \overline{u}}}{{\partial \overline{z}}}} \right)^{2} } \right]^{{\frac{n - 1}{2}}} \left( {\frac{{\partial \overline{u}}}{{\partial \overline{z}}}} \right) + \frac{{\overline{\kappa }^{n + 1} \overline{\psi } }}{{n^{n} }}.$$

Note that in Eq. (8), ${\text{Re}} \left( { = {{\rho U_{HS} H} \mathord{\left/ {\vphantom {{\rho U_{HS} H} {\mu_{aff} }}} \right. \kern-\nulldelimiterspace} {\mu_{eff} }}} \right)$ is the Reynolds number and $\Gamma = \frac{1}{2}{{\left( {\frac{\partial p}{{\partial x}}} \right)} \mathord{\left/ {\vphantom {{\left( {\frac{\partial p}{{\partial x}}} \right)} {\left( {\frac{{m U_{HS}^{n} }}{{H^{n + 1} }}} \right)}}} \right. \kern-\nulldelimiterspace} {\left( {\frac{{m U_{HS}^{n} }}{{H^{n + 1} }}} \right)}}$, which compares the relative strength between the force due to electroosmosis and the pressure force, is termed as the force comparison parameter^31,32^.

### Discretization of non-dimensional momentum transport equation

Here we briefly discuss about the discretization of Eq. (8). For discretizing Eq. (8), we use the forward first-order difference method in time and central second-order difference method in space. Below we write the discretized form of Eq. (8) as:

9 $$\overline{u}_{(i,j)}^{t + 1} = \overline{u}_{(i,j)}^{t} + \frac{{\Delta \overline{t}}}{{\text{Re}}}(F_{1(i,j)}^{t} + F_{2(i,j)} + g_{(i,j)} F^{t}_{3(i,j)} - 2\overline{u}^{t} g_{(i,j)} F_{4} - 2\Gamma ),$$

where,

10 $$\left. \begin{gathered} F_{1(i,j)}^{t} = \left( {\frac{{g_{(i + 1,j)} - g_{(i - 1,j)} }}{{2h_{yz} }}} \right)\left( {\frac{{\overline{u}_{(i + 1,j)}^{t} - \overline{u}_{(i - 1,j)}^{t} }}{{2h_{yz} }}} \right) + \left( {\frac{{g_{(i,j + 1)} - g_{(i,j - 1)} }}{{2h_{yz} }}} \right)\left( {\frac{{\overline{u}_{(i,j + 1)}^{t} - \overline{u}_{(i,j - 1)}^{t} }}{{2h_{yz} }}} \right) \hfill \\ F_{2(i,j)} = \frac{{\overline{\kappa }^{n + 1} \overline{\psi } }}{{n^{n} }} \hfill \\ F_{4} = 2/h_{yz}^{2} \hfill \\ F_{3(i,j)}^{t} = \frac{{\overline{u}_{(i + 1,j)}^{t} + \overline{u}_{(i - 1,j)}^{t} }}{{h^{2}_{yz} }} + \frac{{\overline{u}_{(i,j + 1)}^{t} + \overline{u}_{(i,j - 1)}^{t} }}{{h^{2}_{yz} }} \hfill \\ g_{(y,z)} = \left[ {\left( {\frac{{\partial \overline{u}}}{{\partial \overline{y}}}} \right)^{2} + \left( {\frac{{\partial \overline{u}}}{{\partial \overline{z}}}} \right)^{2} } \right]^{{\frac{n - 1}{2}}} \hfill \\ \end{gathered} \right\}.$$

To eliminate the nonlinearity of Eq. (8), we take into account another function g(y,z). The calculation of g(y,z) is based on the values obtained from the previous iteration.

Now for the discretization of function g(y,z), we employ the central difference discretization scheme for space to obtain the expression as given below.

11 $$g_{(i,j)} = \left[ {\left( {\frac{{ - \overline{u}_{(i - 1,j)} + \overline{u}_{(i + 1,j)} }}{{2h_{yz} }}} \right)^{2} + \left( {\frac{{ - \overline{u}_{(i,j - 1)} + \overline{u}_{(i,j + 1)} }}{{2h_{yz} }}} \right)^{2} } \right]^{{\frac{n - 1}{2}}} .$$

It may be mentioned here that the discretized equations [Eqs. (9)–(11)] are solved numerically as well as by using the spreadsheet analysis tool in order to get the desired results.

In the present study, the value of ${\text{Re}}$, $\Delta \overline{t}$ and $h_{yz}$ is taken as 0.01, $10^{ - 7}$ and $0.02$ respectively, for all the cases in order to achieve an accurate solution with faster convergence. It is assumed that the fluid is initially at rest, i.e., $\overline{u}\left( {\overline{y},\overline{z},0} \right) = 0$. Now, by using the pertinent boundary conditions for potential and velocity, as mentioned in Eqs. (2) and (7) respectively, we obtain the potential and velocity distributions in the flow domain.

## Analytical solution of the transport equations: a limiting case scenario

In this section, we make an attempt to derive the analytical solution for the transport equations in the limiting case pertaining to unsteady, 1-D flow $\left( {{\text{width}} ,\;\;2{\text{W}} > > \;{\text{height}} ,\;2H} \right)$ of a Newtonian fluid $\left( {n = 1} \right)$. Here, we consider the flow to be fully developed $\left( {\frac{\partial u}{{\partial x}} = 0} \right)$ and the axial variation of the ionic concentration to be negligible i.e., $\left( {\frac{\partial \psi }{{\partial x}} = 0} \right)$. We take this attempt for justifying the applicability of the spreadsheet analysis in reproducing the correct results in the context of electrically actuated transport. Under this case, i.e.,$\left( {\frac{\partial }{\partial z}\left( {} \right) \ll \frac{\partial }{\partial y}\left( {} \right)} \right)$, the potential and momentum transport equations reduce to the following.

12 $$\frac{{\partial^{2} \overline{\psi }}}{{\partial \overline{y}^{2} }} = \overline{\kappa }^{2} \overline{\psi },$$

13 $${\text{Re}} \frac{{\partial \overline{u}}}{{\partial \overline{t}}} = - 2\Gamma + \frac{{\partial^{2} \overline{u}}}{{\partial \overline{y}^{2} }} + \overline{\kappa }^{2} \overline{\psi }.$$

Now, we seek solutions for Eqs. (12)–(13) using the boundary conditions mentioned before [cf. Eqs. (2) and (7)] in analytical framework as discussed below.

Considering $\overline{\psi } = \overline{\psi }(\overline{y})$, Eq. (12) can be rearranged as,

14 $$\frac{{\partial^{2} \overline{\psi }}}{{\partial \overline{y}^{2} }} - \overline{\kappa }^{2} \overline{\psi } = 0.$$

Now, on solving this equation we obtain,

$$\overline{\psi } = {\text{A}} \cosh (\overline{\kappa } \, \overline{y}) + {\text{B}} \sinh (\overline{\kappa } \, \overline{y}),$$

where ${\text{A}} ,B$ are constants. By making use of the boundary conditions: $\overline{\psi }(\overline{y} = \pm 1) = - 1$ and $\left. {{{\partial \overline{\psi }} \mathord{\left/ {\vphantom {{\partial \overline{\psi }} {\partial \overline{y}}}} \right. \kern-\nulldelimiterspace} {\partial \overline{y}}}} \right|_{{\overline{y} = 0}} = 0$, we get, ${\text{A}} = - {1 \mathord{\left/ {\vphantom {1 {\cosh \left( {\overline{\kappa }} \right)}}} \right. \kern-\nulldelimiterspace} {\cosh \left( {\overline{\kappa }} \right)}}{\text{ and }}{\text{B}} = 0$.

So, the final form of potential distribution takes the form as:

15 $$\overline{\psi } = - {{\cosh \left( {\overline{\kappa }\overline{y}} \right)} \mathord{\left/ {\vphantom {{\cosh \left( {\overline{\kappa }\overline{y}} \right)} {\cosh \left( {\overline{\kappa }} \right)}}} \right. \kern-\nulldelimiterspace} {\cosh \left( {\overline{\kappa }} \right)}}.$$

For the momentum transport equation [Eq. (13)], we consider the solution in the form as:

16 $$\overline{u}_{sol} = \overline{u}_{CF} + \overline{u}_{PI} .$$

It may be mentioned here that in order to find $\overline{u}_{CF}$, we need an auxiliary equation. The same can be derived from Eq. (13) as given below.

17 $${\text{Re}} \frac{{\partial \overline{u}}}{{\partial \overline{t}}} - \frac{{\partial^{2} \overline{u}}}{{\partial \overline{y}^{2} }} = 0.$$

Now, considering $\overline{u} = Y(\overline{y})T(\overline{t})$, Eq. (17) reduces to the following equation as:

18 $$\frac{Y^{\prime\prime}}{Y} = {\text{Re}} \left( {\frac{T^{\prime}}{T}} \right) = {\text{constant}} = - \alpha^{2} .$$

Now, solving for $Y$ and $T$ we get,

19 $$\left. \begin{gathered} Y = \left\{ {\begin{array}{*{20}l} {A\cos \alpha \overline{y} + B\sin \alpha \overline{y},\;{\text{when}} {(}\alpha \ne {0)}} \\ {D + E\overline{y},{\text{ when (}}\alpha { = 0)}} \\ \end{array} } \right. \hfill \\ T = \left\{ {\begin{array}{*{20}l} {F\exp \left( {\frac{{ - \alpha^{2} \overline{t}}}{{\text{Re}}} \, } \right){\text{, when (}}\alpha \ne {0)}} \\ {G,{\text{ when (}}\alpha = {0)}} \\ \end{array} } \right. \hfill \\ \end{gathered} \right\}.$$

Therefore, the expression of $\overline{u}_{CF}$ becomes as follows:

20 $$\, \overline{u}_{CF} = (A\cos \alpha \overline{y} + B\sin \alpha \overline{y})F\exp \left( {\frac{{ - \alpha^{2} \overline{t}}}{{\text{Re}}}} \right) + (D + E\overline{y})G = (P\cos \alpha \overline{y} + Q\sin \alpha \overline{y})\exp \left( {\frac{{ - \alpha^{2} \overline{t}}}{{\text{Re}}}} \right) + R\overline{y} + S.$$

Note that in Eq. (20), $P\left( { = AF} \right)$, $Q\left( { = BF} \right)$, $R\left( { = EG} \right)$ and $S\left( { = DG} \right)$ are all arbitrary constants.

Now, in an effort to obtain $\overline{u}_{PI}$, Eq. (13) can be reduced to the following,

21 $$(D_{1}^{2} - {\text{Re}} D_{2} )\overline{u}_{PI} = 2\Gamma - \overline{\kappa }^{2} \overline{\psi };\;{\text{where}}\,D_{1} = {\partial \mathord{\left/ {\vphantom {\partial {\partial \overline{y}}}} \right. \kern-\nulldelimiterspace} {\partial \overline{y}}}{\text{ and }}D_{2} = {\partial \mathord{\left/ {\vphantom {\partial {\partial \overline{t}}}} \right. \kern-\nulldelimiterspace} {\partial \overline{t}}}.$$

Hence, we get the following,

22 $$\overline{u}_{PI} = \frac{1}{{D_{1}^{2} - {\text{Re}} D_{2} }}(2\Gamma ) - \frac{1}{{D_{1}^{2} - {\text{Re}} D_{2} }}(\overline{\kappa }^{2} \overline{\psi }).$$

Now, we can write that

23 $$\frac{1}{{D_{1}^{2} - {\text{Re}} D_{2} }}(2\Gamma ) = \frac{1}{{D_{1}^{2} }}(2\Gamma ) = \overline{y}^{2} \Gamma .$$

24 $${\text{And}}, \left. \begin{aligned} \frac{1}{{D_{1}^{2} - {\text{Re}} D_{2} }}(\overline{\kappa }^{2} \overline{\psi }) & = \overline{\kappa }^{2} \frac{1}{{D_{1}^{2} - {\text{Re}} D_{2} }}\left( { - \frac{{\cosh (\overline{\kappa } \, \overline{y})}}{{\cosh (\overline{\kappa })}}} \right) \\ & = - \frac{{\overline{\kappa }^{2} }}{{\cosh (\overline{\kappa })}}\frac{1}{{D_{1}^{2} - {\text{Re}} D_{2} }}(e^{{\overline{k} \, \overline{y}}} + e^{{ - \overline{k} \, \overline{y}}} ) = - \frac{{\cosh (\overline{\kappa } \, \overline{y})}}{{\cosh (\overline{\kappa })}} = \overline{\psi } \\ \end{aligned} \right\}.$$

Now, from Eqs. (22)–(24), we can write the following as:

25 $$\overline{u}_{PI} = \overline{y}^{2} \Gamma - \overline{\psi }.$$

Note that upon substituting Eqs. (20) and (25) in Eq. (16), we get the following as:

26 $$\overline{u} = (P\cos \alpha \overline{y} + Q\sin \alpha \overline{y})\exp \left( {\frac{{ - \alpha^{2} \overline{t}}}{{\text{Re}}}} \right) + R\overline{y} + S + \overline{y}^{2} \Gamma - \overline{\psi }.$$

Next, we need to find the constants $P$, $Q$, $R$, $S$ and $\alpha$ by making use of different boundary conditions pertinent to this analysis. Applying boundary conditions $\overline{u}(\overline{y} = 1,\overline{t}) = 0$ and $\overline{\psi }(\overline{y} = \pm 1) = - 1$, we get the following equation as:

27 $$(P\cos \alpha + Q\sin \alpha )\exp \left( {\frac{{ - \alpha^{2} \overline{t}}}{{\text{Re}}}} \right) + R + S + \Gamma + 1 = 0.$$

And, applying another set of boundary conditions $\overline{u}(\overline{y} = - 1,\overline{t}) = 0$ and $\overline{\psi }(\overline{y} = \pm 1) = - 1$, we get the below-given expression.

28 $$(P\cos \alpha - Q\sin \alpha )\exp \left( {\frac{{ - \alpha^{2} \overline{t}}}{{\text{Re}}}} \right) - R + S + \Gamma + 1 = 0.$$

Now, upon subtracting Eq. (28) from Eq. (27), we get the following.

29 $$(Q\sin \alpha )\exp \left( {\frac{{ - \alpha^{2} \overline{t}}}{{\text{Re}}}} \right) + R(1) = 0.$$

Now, we calculate the Wronskian of $\exp \left( {\frac{{ - \alpha^{2} \overline{t}}}{{\text{Re}}}} \right)$ and 1 with respect to $t$ as given below.

$W\left[ {\exp \left( {{{ - \alpha^{2} \overline{t}} \mathord{\left/ {\vphantom {{ - \alpha^{2} \overline{t}} {\text{Re}}}} \right. \kern-\nulldelimiterspace} {\text{Re}}}} \right),1} \right](t) \ne 0$, so $\exp \left( {{{ - \alpha^{2} \overline{t}} \mathord{\left/ {\vphantom {{ - \alpha^{2} \overline{t}} {\text{Re}}}} \right. \kern-\nulldelimiterspace} {\text{Re}}}} \right)$ and 1 are linearly independent. Now, by using the condition of the theorem of linear independence in Eq. (29), we get the following as:

30 $$\left. {\begin{array}{*{20}c} {Q \times \sin \alpha } \\ R \\ \end{array} } \right\} = 0.$$

Upon substituting the second condition of Eq. (30) in Eq. (26), we can write the below given form.

31 $$\overline{u} = (P\cos \alpha \overline{y} + Q\sin \alpha \overline{y})\exp \left( {\frac{{ - \alpha^{2} \overline{t}}}{{\text{Re}}}} \right) + S + \overline{y}^{2} \Gamma - \overline{\psi }.$$

We further use the boundary condition $\overline{u}(\overline{y} = 0,\overline{t} = 0) = 0$ in Eq. (31), and obtain the following.

32 $$P + S + \frac{1}{{\cosh (\overline{\kappa })}} = 0 \, \left[ { \, \because \overline{\psi }(\overline{y} = 0) = - \frac{1}{{\cosh (\overline{\kappa })}}} \right].$$

Also, on applying the boundary condition $\overline{u}(\overline{y} = 1,\overline{t} = 0) = 0$ and $\overline{\psi }(\overline{y} = 1,\overline{t} = 0) = - 1$ and using first condition of Eq. (30) in Eq. (31), we get the expression as written below.

33 $$P\cos \alpha + S + \Gamma + 1 = 0.$$

Now, we compare Eqs. (32) and (33) to obtain as follows: If $\cos \alpha = 1$, then $\Gamma + 1 = \left\{ {\cosh (\overline{\kappa })} \right\}^{ - 1}$, which is practically impossible as $\Gamma$ and $\overline{\kappa }$ are independent of each other. Thus, what follows is that $\cos \alpha \ne 1$, which implies $\sin \alpha \ne 0$. Now, from Eq. (30) we get, $Q\sin \alpha = 0$, which implies $Q = 0$. We next substitute $Q = 0$ in Eq. (31) and get the below written expression.

34 $$\overline{u} = (P\cos \alpha \overline{y})\exp \left( {\frac{{ - \alpha^{2} \overline{t}}}{{\text{Re}}}} \right) + S + \overline{y}^{2} \Gamma - \overline{\psi }.$$

We next apply the boundary condition $\overline{u}\left( {\overline{y} = 1,\overline{t} = 0} \right) = 0$ and $\overline{\psi }\left( {\overline{y} = 1,\overline{t} = 0} \right) = - 1$ in Eq. (34) essentially to find out constants $P$ and $S$. The obtained form is written below.

35 $$(P\cos \alpha )\exp \left( {\frac{{ - \alpha^{2} \overline{t}}}{{\text{Re}}}} \right) + (S + \Gamma + 1) = 0.$$

Now, as $\exp \left( {{{ - \alpha^{2} \overline{t}} \mathord{\left/ {\vphantom {{ - \alpha^{2} \overline{t}} {\text{Re}}}} \right. \kern-\nulldelimiterspace} {\text{Re}}}} \right)$ and 1 are linearly independent, it suffices to the following expression.

36 $$\left. {\begin{array}{*{20}c} {P \times \cos \alpha } \\ {S + \Gamma + 1} \\ \end{array} } \right\} = 0.$$

Now, $P = 0$ leads to the form of velocity as $\overline{u} = S + \overline{y}^{2} \Gamma - \overline{\psi }$, and then $\overline{u}$ has no time-dependence. Thus, this is not possible since the problem considered in this study is unsteady one. Therefore, $\cos \alpha = 0$, and gives $\alpha = (2n - 1)\pi /2 \,$, where ${\text{n}} \in {\mathbf{\mathbb{Z}}}$. Now from Eq. (32), we get,

37 $$P = - \left( {S + (\cosh (\overline{\kappa }))^{ - 1} } \right).$$

Thus, we obtain the final form of velocity as:

38 $$\overline{u} = (P\cos \alpha \overline{y})\exp \left( {\frac{{ - \alpha^{2} \overline{t}}}{{\text{Re}}}} \right) + S + \overline{y}^{2} \Gamma - \overline{\psi },$$

where, $$S = - (\Gamma + 1)$$, $$\alpha = (2n - 1)\pi /2{\text{ where n}} \in {\mathbf{\mathbb{Z}}}$$ and $$P = - \left( {S + (\cosh (\overline{\kappa }))^{ - 1} } \right)$$.

## Selection of model parameters

Before describing the results, we here mention numerical value of several parameters as considered in this analysis. The chosen values of different parameters are $\overline{\kappa } = 20$, $n = 0.8,\,\,1\;{\text{and}} \;1.2$, $\Gamma = 0,\,\,1\;{\text{and}} \;2$. Although the present analysis focuses on the flow dynamics of power law non-Newtonian fluids, we consider $n = 1$ in depicting results for some cases for two different reasons. First, results pertaining to $n = 1$ are considered to validate the modelling framework employed in this work with our own analytical results from the perspective unsteady electrically actuated transport as well as with the results reported by Zhao et al.^28^. Second, pertinent to this analysis, the depicted variation for $n = 1$ is of significance as to isolate the rheological effect on the underlying flow dynamics.

## Electroosmotic flow analysis using microsoft excel

### Microsoft excel configuration

Here, we present the solution strategies of the discretized form of the governing equations using spreadsheet analysis tool in Microsoft Excel. The entire computational domain is divided into $100\, \times \,100$ grids. Each cell in the Excel sheet represents one grid. The grey-colored cells represent the boundary conditions. In the present study, no-slip boundary condition is taken into consideration for the velocity i.e., $\overline{u} = 0$ and $\overline{\psi } = - 1$ for potential.

The spreadsheet contains seven panels. They are described as follows.

- First panel—It computes the distribution of potential $\overline{\psi }$ required for the calculation of the velocity profile.
- Second panel—It computes the velocity of the fluid at the time $\overline{t}$ i.e., $\overline{u}^{{(\overline{t},\overline{y},\overline{z})}}$.
- Third panel—It computes the velocity of the fluid at the time $\overline{t} + \Delta \overline{t}$ i.e., $\overline{u}^{{(\overline{t} + \Delta \overline{t},\overline{y},\overline{z})}}$.
- Fourth panel—It computes the function $g_{(y,z)}$.
- Fifth panel—It computes the function $F_{1(y,z)}^{{}}$.
- Sixth panel—It computes the function $F_{2(y,z)}^{{}}$.
- Seventh panel—It computes the function $F_{3(y,z)}^{{}}$.

**Figure 2:**
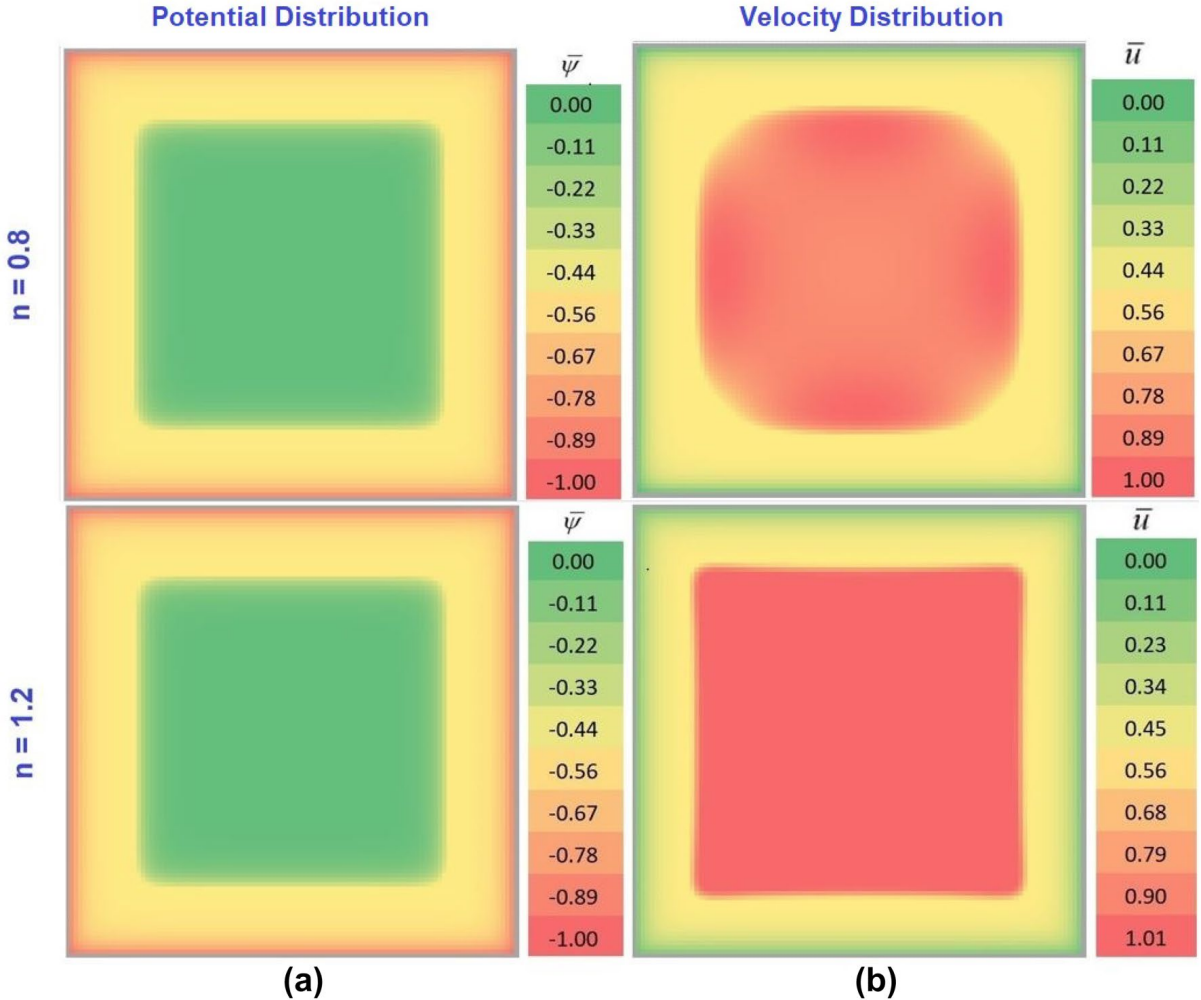
Contour plot of non-dimensional (**a**) potential distribution and (**b**) velocity distribution for $\overline{\kappa } = 20$, and $\Gamma = 0$ for $n = 0.8$ and $n = 1.2$ in the top and the bottom panel respectively using Microsoft Excel.

All the panels are color-coded. Figure 2a,b represent the potential and velocity distribution contours respectively, obtained from spreadsheet analysis for $\overline{\kappa } = 20$, $\Gamma = 0$. While the variation shown in the top panel conforms to $n = 0.8$, the same at the lower panel is obtained for $n = 1.2$. Quite notably, we do not find any noticeable difference in the potential distribution with a change in $n$ as witnessed in Fig. 2a. Nevertheless, distinct difference in the velocity distribution with $n$ as observed in Fig. 2b is attributed to the fluid rheological effect. The magnitude of potential is found to be maximum ($\overline{\psi } = - 1$) near the walls due to the development of the electrical double layer (EDL) and minimum ($\overline{\psi } = 0$) at the center of the microchannel. The velocity is found to be minimum ($\overline{u} = 0$) at the walls due to zero tangential velocity along the wall. We have also used color bars at the right of Fig. 2a,b in order to facilitate easy understanding of the readers.

### Iteration

**Figure 3:**
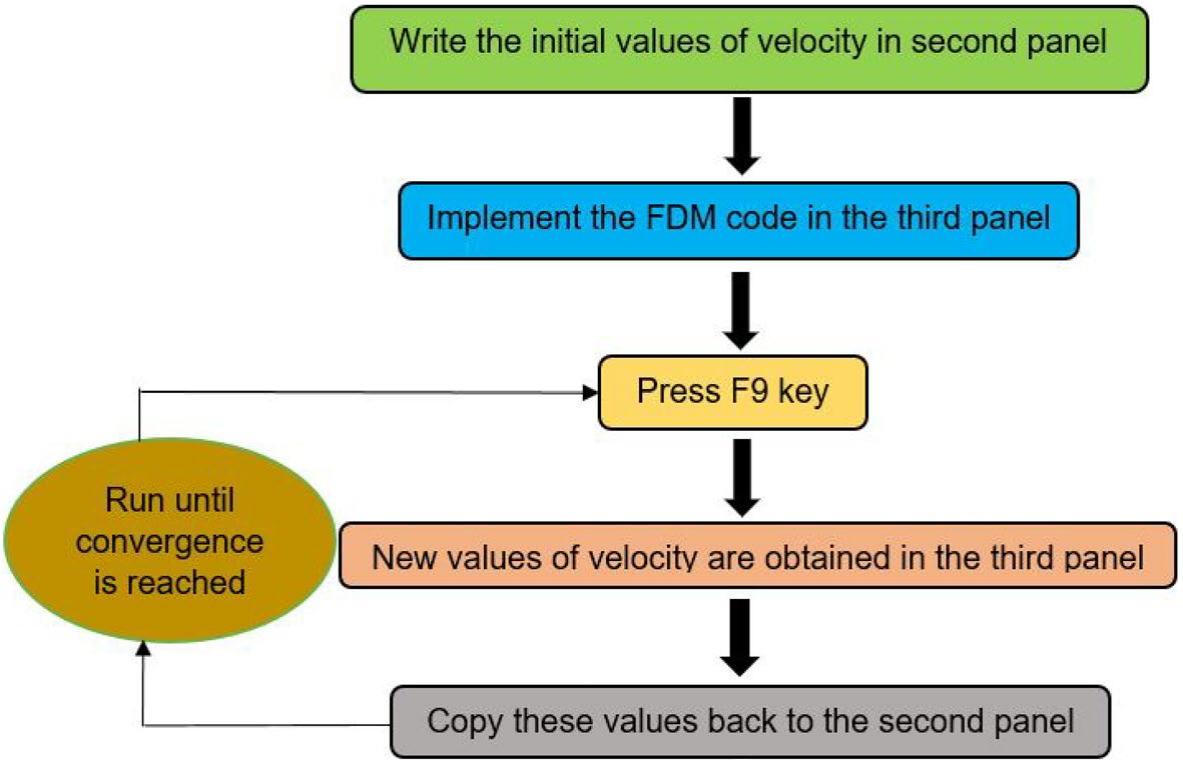
Schematic diagram describing the implementation procedure of the numerical scheme in Microsoft Excel.

There is no requirement for iteration inside a single time instant because the numerical method employed to solve Eq. (9) is first-order in time. The maximum number of iterations is therefore set to 1. According to this plan, one time step must be completed without requiring any additional iterations. To accomplish this, follow the steps below: click "File—Settings—Computation options," then click "Enable iterative calculation," then "Maximum Iterations" should be set to 1. The F9 key on the keyboard is used to perform successive iterations that stand in for various temporal instants. The implementation procedure of the numerical scheme is depicted in a schematic diagram for the readers' in-depth knowledge, as schematically shown in Fig. 3. The Supporting Information part of the article “Electroosmotic start-up microflows.xlsx” contains the detailed steps.

## Model characterization and validation

### Validation of spreadsheet solution with the analytical results

**Figure 4:**
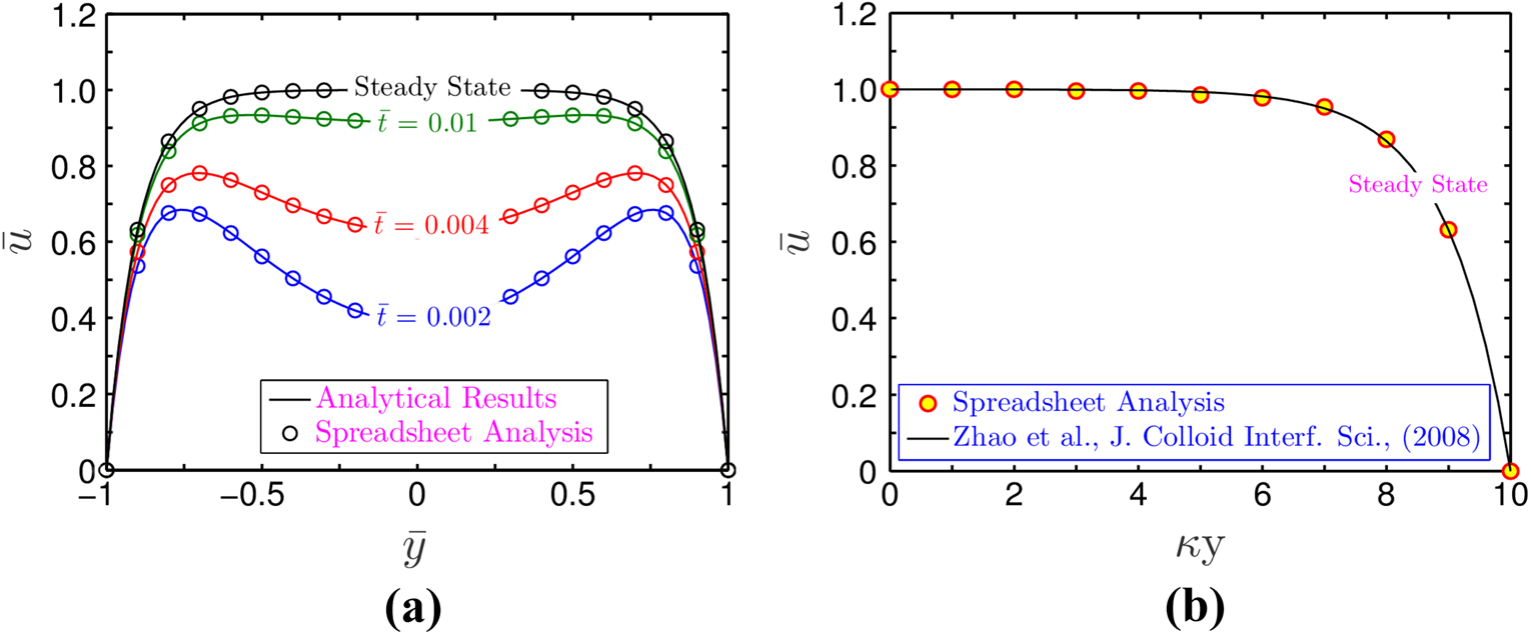
(**a**) Plots depict velocity profile at different instances of time at $z = 0$ and (**b**) Plots depict steady state velocity profile at $z = 0$. The parameters used for these variations are $\overline{\kappa } = 10$, and $\Gamma = 0$ for $n = 1$. The analytical solutions are represented by solid lines, whereas the spreadsheet analysis tool-derived solutions are shown by markers.

We try to thoroughly benchmark the modelling framework used for this investigation. In doing so, we adhere to the dual benchmarking technique and go over the following in detail. First, the velocity profile at different temporal instants obtained by solving Eq. (38), is depicted in Fig. 4a and is compared with that of spreadsheet solution for a limiting case considering unsteady, 1-D flow $\left( {2{\text{W}} > > 2H} \right)$. Second, the analytical solution of the steady state scenario produced from the spreadsheet analysis tool is also validated with the analytical solution of Zhao et al.^28^ in Fig. 4b. The other parameters used for these validation plots are $\overline{\kappa } = 10$, $\Gamma = 0$ and $n = 1$. It is worth adding here that the results reported by Zhao et al.^28^ conform to the analytical solution of steady state electroosmotic velocity distribution in a parallel plate channel. As witnessed in Fig. 4a,b, the results obtained from spreadsheet analysis are in good agreement with both the analytical solutions. The effectiveness of the spreadsheet analysis used in this work for capturing the intended flow physics is supported by this observation.

### Comparison of spreadsheet results with the analytical solutions: Validation from the perspective of fluid rheology

**Figure 5:**
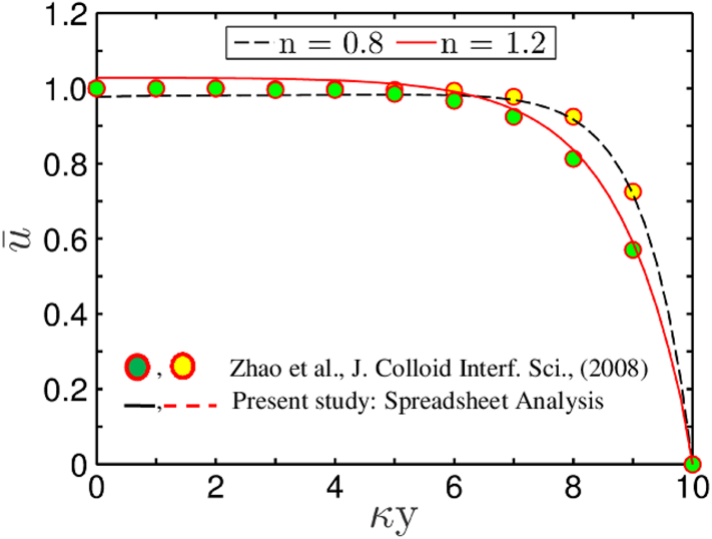
Plots depict validation of the present CFD code with that of Zhao et al.^28^ using Spreadsheet analysis tool. The solid lines depicts the velocity half profile obtained from the present study, while, the markers represent the analytical solution obtained by Zhao et al.^28^. The parameters used for validation are $\overline{\kappa } = 10$, and $\Gamma = 0$ for $n = 0.8$ and $n = 1.2$. The velocity profile is obtained at $z = 0$ for both cases.

In this section, we take into account the effect of fluid rheology for the validation of the present model as graphically demonstrated in Fig. 5. For the plots depicted in Fig. 5, we take the parameters as $\overline{\kappa } = 10$, $\Gamma = 0$, while the shear-thinning $\left( {n = 0.8} \right)$ and shear-thickening $\left( {n = 1.2} \right)$ effects are considered. It is observed that the steady-state velocity profile obtained from both the numerical methods and spreadsheet analysis tool closely matches with the analytical results of Zhao et al.^28^. This aspect signifies the creditability of the numerical scheme employed in this study.

## Results and discussion

### Potential distribution

**Figure 6:**
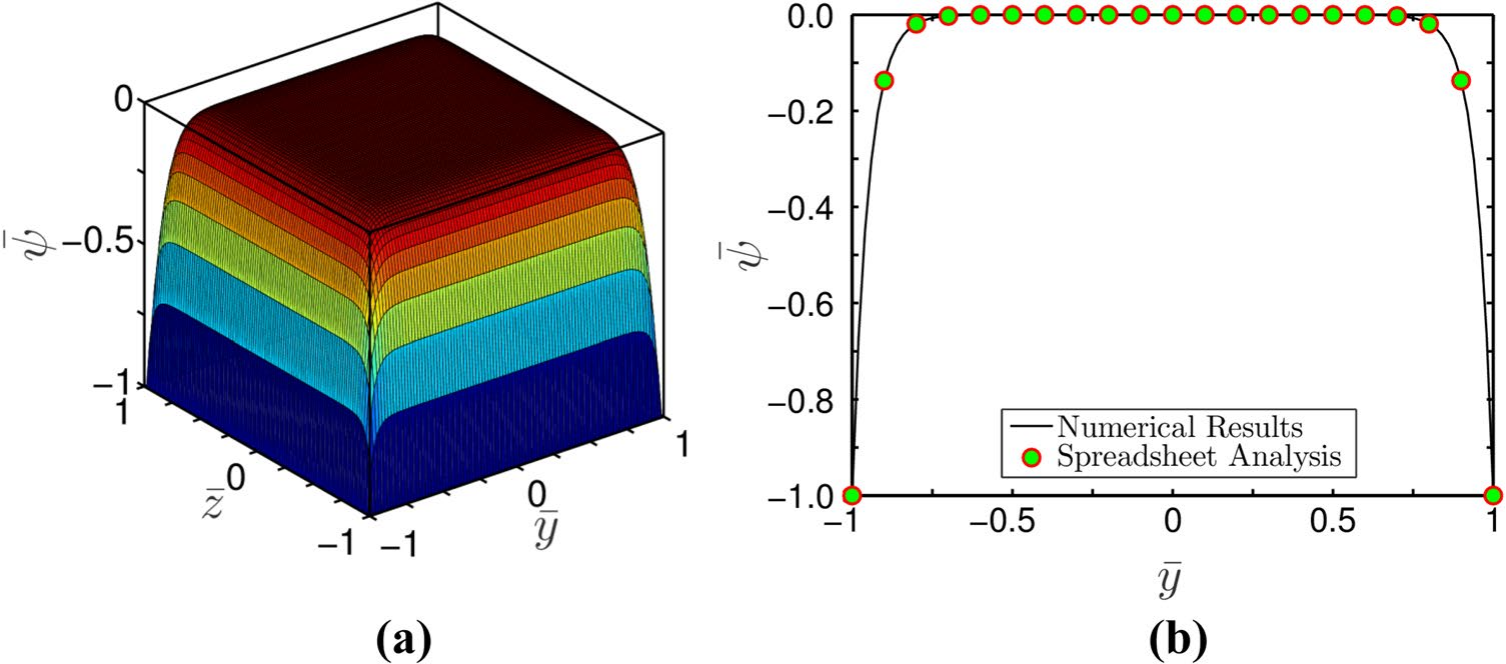
Plots depict potential distribution profile for $\overline{\kappa } = 20$: (**a**) Three-dimensional potential distribution using Spreadsheet analysis tool, and (**b**) Potential distribution profile at $z = 0$ using both MATLAB and Spreadsheet analysis tool.

We initiate our discussion with Fig. 6a,b, which shows the distribution of potential in the square domain for $\overline{\kappa } = 20$. As was previously said, when an electrolytic solution is present, the walls of the micro-channel are subjected to net charge, which causes the creation of a (EDL) Electric Double Layer. As a result, potential is found to be zero at the centre of the channel and its magnitude increases towards the walls. This phenomenon results in a plug-like profile as observed in Fig. 6a,b.

### Transient flow hydrodynamics

In this section, we show how power law non-Newtonian fluids start to flow dynamically under the influence of a field in a microfluidic channel. For obtaining the results, we consider both the applied pressure gradient and electroosmotic effect. For each case, we compare the results obtained from numerical methods and spreadsheet analysis considering different values of $\Gamma$, and $n$. We discuss them systematically in the forthcoming sections.

**Figure 7:**
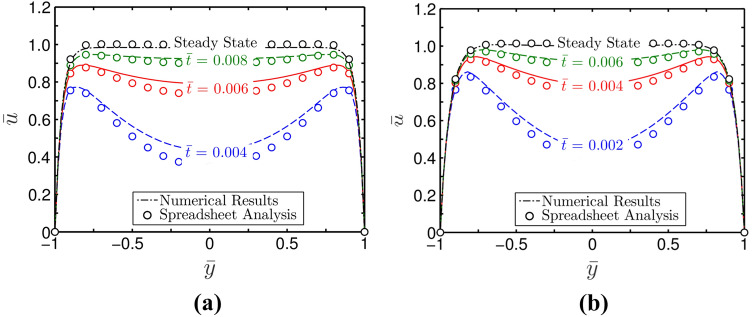
Plots depict velocity profile at different instances of time at $z = 0$. The parameters used for these temporal variations are $\overline{\kappa } = 20$, and $\Gamma = 0$ for (**a**) $n = 0.8$ and (**b**) $n = 1.2$. The MATLAB solutions are represented by solid lines, whereas the spreadsheet analysis tool-derived solutions are shown by markers.

As seen in Fig. 7a,b, which depict the temporal variation of flow velocity for shear-thinning $\left( {n = 0.8} \right)$ and shear-thickening $\left( {n = 1.2} \right)$ fluids respectively, initially the velocity profile exhibits its maxima near the wall of the microchannel, and depression is found at the centre of the channel. We consider results obtained from both numerical method and spreadsheet analysis in plotting the variations depicted in Fig. 7a,b. The parameters used for this plot are $\overline{\kappa } = 20$ and $\Gamma = 0$. A plug-type velocity profile is obtained in the steady state as the depression that initially appeared on the velocity profile flattens over time. From the depicted variations in Fig. 7a,b, the maximum value of velocity is found at the center of the channel. A fairly accurate match between the results obtained from numerical calculations and spreadsheet analysis as verified in Fig. 7 indicates the efficacy of spreadsheet analysis in obtaining complex details of the start-up electrokinetic flow of non-Newtonian fluids.

**Figure 8:**
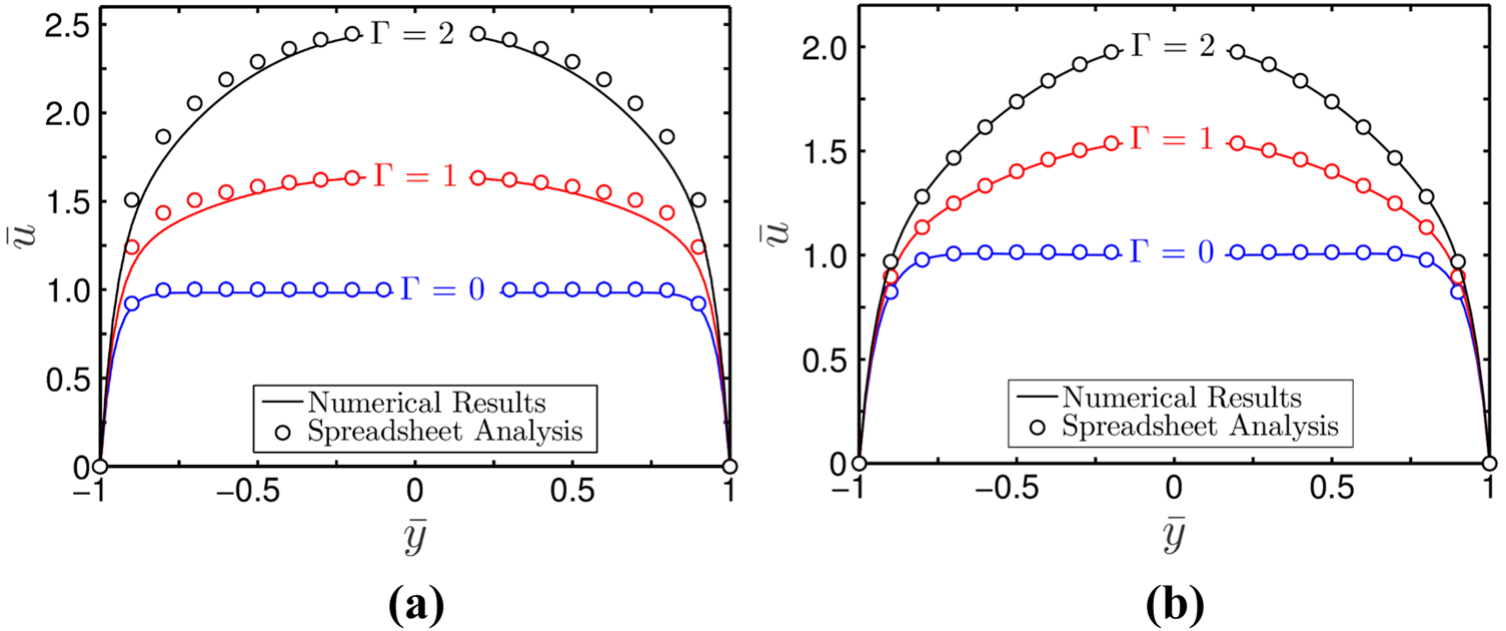
Plots depict velocity profile for different values of $\Gamma$ at $z = 0$. The other parameters are taken as $\overline{\kappa } = 20$ for (**a**) $n = 0.8$ and (**b**) $n = 1.2$. The MATLAB solutions are represented by solid lines, whereas the spreadsheet analysis tool-derived solutions are shown by markers.

Now, we discuss in Fig. 8a,b the variation of steady-state velocity profile for shear-thinning $\left( {n = 0.8} \right)$ and shear-thickening $\left( {n = 1.2} \right)$ fluids respectively, obtained for different values of $\Gamma =$(0, 1, and 2) and $\overline{\kappa } = 20$. The MATLAB solutions are represented by solid lines, whereas the spreadsheet analysis tool-derived solutions are shown by markers. It is found that with the increase in $\Gamma$, the maximum value of velocity increases. It may be mentioned here that $\Gamma$ being the force comparison parameter, compares the impact of force due to applied pressure gradient to the electroosmotic effect on the underlying transport. Thus, following this definition, for $\Gamma$ > 1, electroosmotic effect becomes less effective than the applied pressure gradient on the transport. While for $\Gamma$ < 1, forcing due to applied pressure gradient becomes less effective than electroosmotic effect of the underlying transport, and for $\Gamma$ = 1, both of these two forces have an equal effect on the flow dynamics. Therefore, it is found that as $\Gamma$ increases, the velocity profile tends to become more parabolic in nature. However, for the lesser values of $\Gamma$, the velocity profile showing similarity with the electroosmotic velocity exhibits plug-like in nature. This observation once more signifies the capability of spreadsheet analysis in accurate description of flow field accounting for the applied field effect in the analysis.

**Figure 9:**
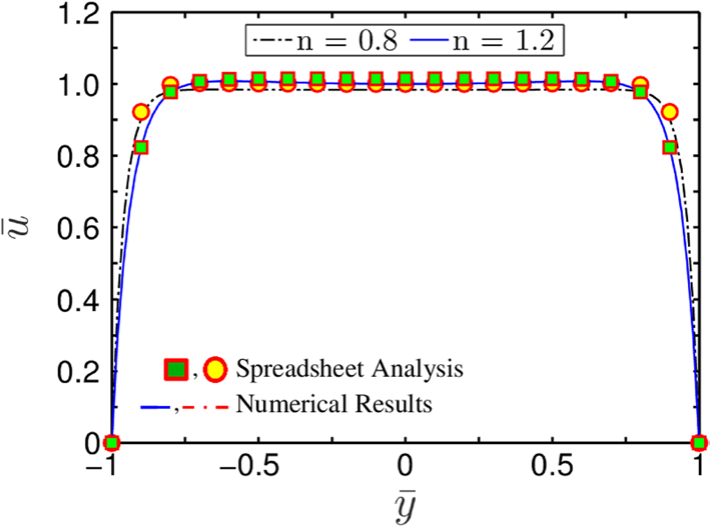
Plots depict velocity profile for different values of $n$ at $z = 0$. The other parameters are taken as $\overline{\kappa } = 20$, and $\Gamma = 0$. The MATLAB solutions are represented by solid lines, whereas the spreadsheet analysis tool-derived solutions are shown by markers.

**Figure 10:**
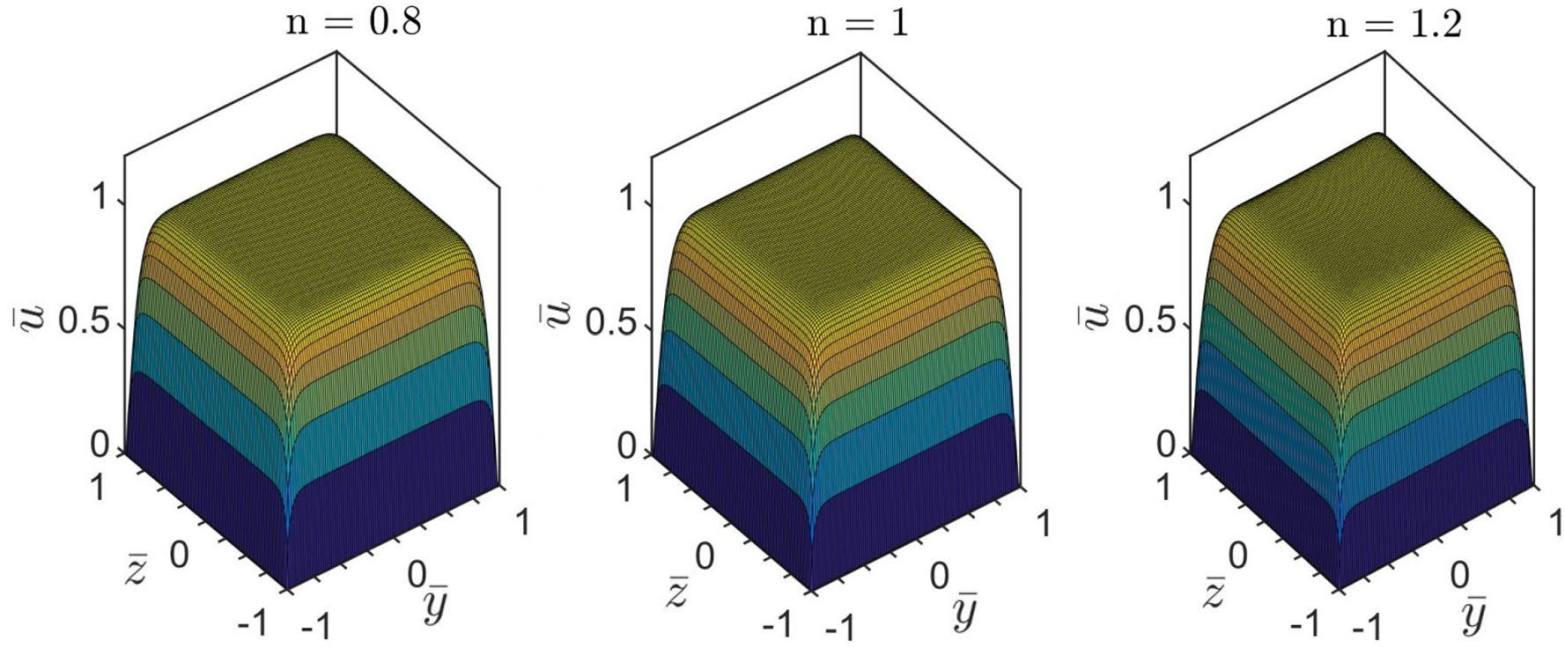
Plots depict three-dimensional velocity distribution for different values of $n$ at $\overline{\kappa } = 20$, and $\Gamma = 0$ using Spreadsheet analysis tool.

Next, we make an attempt, in Figs. 9 and 10, to denote the difference in steady-state velocity distribution profile for two different values of $n = 0.8$ and $n = 1.2$, obtained at $\overline{\kappa } = 20$ and $\Gamma = 0$. It is found that the velocity profile gets more flattened with decreasing the value of $n$. We attribute this finding as follows. With the increase in the value of $n$, the apparent viscosity of the fluid increases. This is because, the influence of electroosmotic body force being delivered on the fluid reduces as the apparent viscosity of the fluid increases. Albeit the strength of electroosmotic forcing remains constant for a given set of parameters, its realisation on the underlying transport phenomenon becomes less effective with increasing the value of $n$, primarily due to flow's increased viscous resistance. In particular, upon experiencing a relatively lesser resistance, shear-thinning fluid $\left( {n = 0.8} \right)$ attains maximum flow velocity nearer to the walls of the channel. Also, as seen from Figs. 9 and 10, the maximum value of the flow velocity at the center of the microchannel is approximately equal in all cases. Looking at the variation represented in Fig. 9, it may be inferred that spreadsheet analysis tool is equally effective in capturing the intuitive flow physics quite accurately.

### Discussion on net throughput

**Figure 11:**
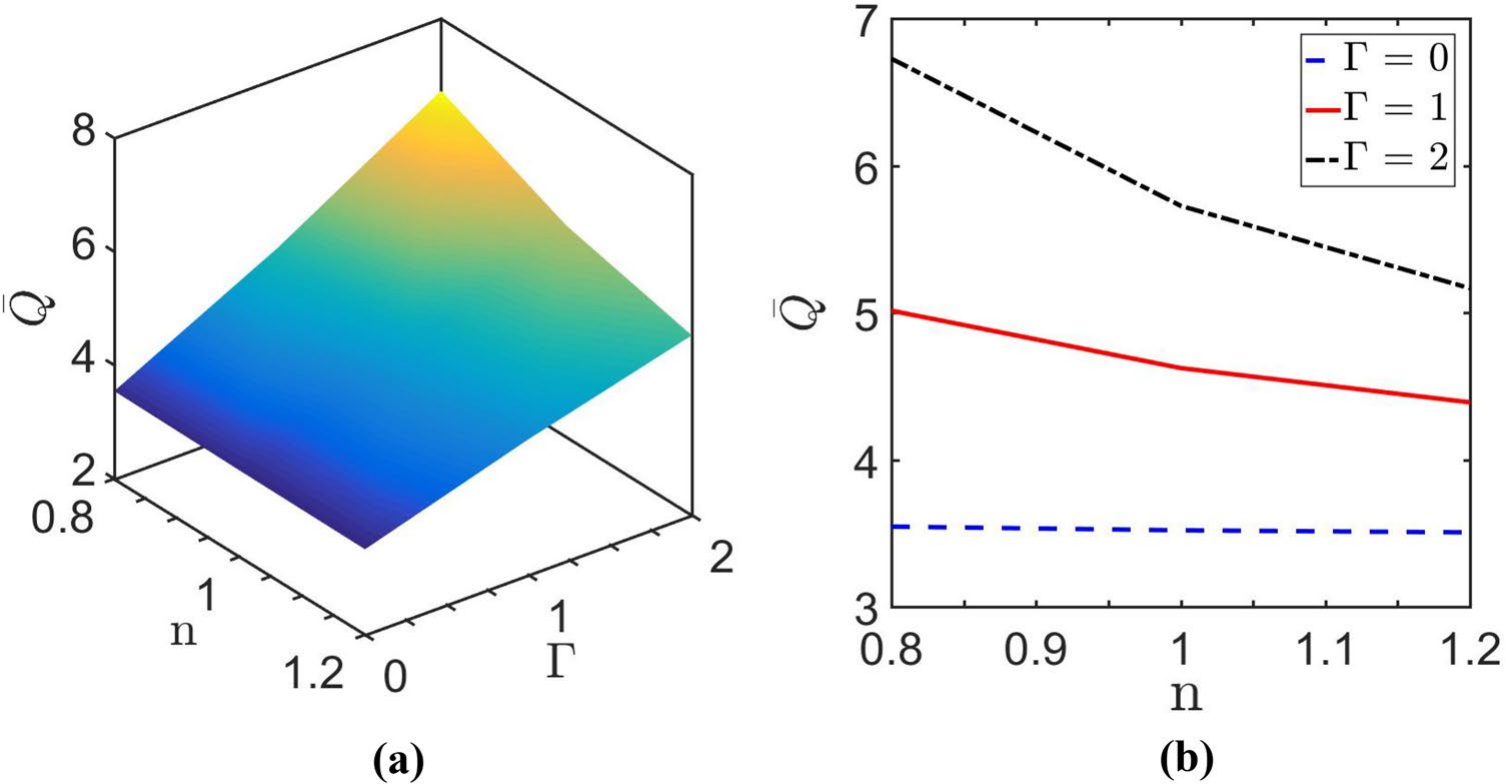
Plots depict volume flow rate $\left( {\overline{Q}} \right)$ variation: (**a**) variation is shown in $n - \Gamma$ plane; (**b**) variation is shown with a change in $n$ for $\overline{\kappa } = 20$ and different values of $\Gamma$.

In order to complete the discussion, we look into the variation of net throughput, commonly named as volumetric flow rate, for different values of $\Gamma$ and $n$ as depicted in Fig. 11. The net throughput is an important parameter to be considered for the optimal design of microfluidic devices. In microfluidic devices/systems, the purpose of embedding various types of annexations ultimately aim at the improvement of the net throughput flow rate as well as its precise control by several means, essentially to fulfil the demanding need of MEMS and μTAS. In the context of present fluidic configuration, the expression for the dimensionless volume flow rate is given as:

$$\overline{Q} = \int_{ - 1}^{1} {\int_{ - 1}^{1} {\overline{u} (\overline{z} ,\overline{y} )d\overline{z} d\overline{y} } } .$$

Figure 11a plots the variation of net throughput in $\Gamma - n$ plane and obtained for $\overline{\kappa } = 20$. With the increase in $\Gamma$, pressure gradient increases. Hence, the effective net throughput in the channel increases significantly as the pressure gradient's magnitude increases because it entirely acts over lateral aspect of the microchannel. As a result, the volume flow rate increases with the increase in $\Gamma$, which is also shown in Fig. 11b. Also, it is found that the net throughput enhances with decreasing the value of $n$. This observation is better understood in Fig. 11b. This is because the viscous resistance of the shear-thickening fluid ($n > 1$) is more than that of the shear-thinning fluid ($n < 1$), and this higher viscous resistance results in a reduction in net throughput of shear-thickening $\left( {n > 1} \right)$ fluid.

## Conclusion

Using analytical methodology and numerical analysis, we have examined the electroosmotic unsteady flow of non-Newtonian fluids in a microfluidic channel having square cross-section. In keeping with the main goal of this study, we have appropriately discussed the solution methodology of the transport equations by incorporating their discretized forms into the Spreadsheet tool and deliberated on the variation of electrostatic potential as well as flow velocity in the selected fluidic domain for a number of cases. Also, we have demonstrated an analytical method for the solution of an unsteady, electrically actuated flow in the limiting case and used analytical results to compare the solutions obtained from Spreadsheet analysis. We have demonstrated that the spreadsheet tool is effective at capturing the crucial flow mechanics in both the initial transience and steady state conditions under the modulation of the electric double layer phenomenon by demonstrating one-to-one comparison between the solutions obtained from Spreadsheet analysis vis-à-vis corresponding numerical results for the cases pertaining to this analysis. We anticipate that the shown deductions from this analysis will attest to the capability of readily accessible Spreadsheet tools in efficiently and affordably resolving computationally demanding challenges.

## Supplementary Information

Supplementary Information 1.

## Data Availability

All data generated or analysed during this study are included in this article and its supplementary information files (Electroosmotic start-up microflows.xlsx).
